# An exploratory study on the role of serum fatty acids in the short-term dietary therapy of gingivitis

**DOI:** 10.1038/s41598-022-07989-5

**Published:** 2022-03-07

**Authors:** Anne B. Kruse, Maximilian Gärtner, Kirstin Vach, Dirk Grueninger, Stefanie A. Peikert, Petra Ratka-Krüger, Christian Tennert, Johan P. Woelber

**Affiliations:** 1 Department of Operative Dentistry and Periodontology, Faculty of Medicine, University of Freiburg, Hugstetter Str. 55, 79106 Freiburg, Germany; 2 Department of Medical Biometry and Medical Informatics, Faculty of Medicine, University of Freiburg, Stefan-Meier-Strasse 26, 79104 Freiburg, Germany; 3 Centre of Laboratory Diagnostics MVZ Clotten, Merzhauser Str. 112 a, 79100 Freiburg, Germany; 4 Department of Restorative, Preventive and Pediatric Dentistry, University of Berne, Freiburgstrasse 7, 3010 Berne, Switzerland

**Keywords:** Randomized controlled trials, Gingivitis, Nutrition

## Abstract

A previous randomised controlled trial showed that an anti-inflammatory diet (AID) significantly reduced gingival inflammation despite constant plaque values. This exploratory study investigated the role of serum fatty acids in relation to the observed clinical effects. Therefore, data of thirty participants with gingivitis, following either a pro-inflammatory dietary pattern (PID) rich in saturated fat, omega 6 fatty acids, and refined carbohydrates or an AID for 4 weeks, were correlated with corresponding serum samples for a variety of fatty acids. Changes in the fatty acid profile and effects on clinical periodontal parameters were analysed. Results showed that the polyunsatured:saturated fatty acids ratio (PUFA:SFA ratio) and nervonic acid level were significantly higher in the AID group than in the PID group at the end of the study. Significant intragroup differences were seen only in the AID group. Diverse fatty acids showed heterogeneous relations to clinical parameters. This study demonstrated that the serum fatty acid profile was not fundamentally associated with the clinical gingivitis-lowering effects of an AID in short-term, although some fatty acids showed individual relations to clinical parameters with respect to inflammation. Hence, short-term effects of dietary therapy on gingivitis may be rather based on carbohydrate-related effects and/or micronutrients.

## Introduction

Periodontitis is one of the most common chronic diseases of mankind. In 2015, the Global Burden of Disease Study estimated that there were approximately 570 million cases of severe periodontitis worldwide^1^. Gingivitis is a basic prerequisite for the development of periodontitis^2^. While for several decades, the destruction of bacterial biofilms has been the major focus of periodontal therapy and is still the proposed as the treatment of choice^3^, there is growing evidence that dental plaque may not be the true cause of gingivitis^4^. In this context, a landmark study conducted under stone-age conditions (simulated from archeological findings between 4000- and 3500 BC) found that gingival inflammation decreased due to stone-age dietary pattern, even in the absence of any plaque control measures and higher dental plaque values^5^. Available nutrition during this stone-age setting included whole grains from resident grain like barley, wheat and spelt, herbs, honey, milk, salt and meat from goats and hens. The authors concluded that the relation between plaque and gingival inflammation might not be valid under a diet omitting processed foods, in particular refined sugars^5^. Meanwhile, it has been found that the gingivitis-associated biofilm (predominantly represented by anaerobic, proteolytic bacteria) is highly depended on inflammatory processes resulting in a higher exudation of gingival crevicular fluid^6^. Due to these nutritional dependencies these bacteria can be considered as inflammophilic^7^. Therefore, current therapeutic approaches in the context of host modulation therapy (HMT) are aimed at treating inflammation^4^.

In HMT life-style modifications, such as smoking cessation and dietary interventions, are important as causal approaches^8,9^. In addition to its benefit for general health, there is evidence that an anti-inflammatory diet (AID) can significantly decrease gingival inflammation, as found as primary outcome of a previous study, which was accompanied by a weight loss of 1.5 kg per participant after a 4-week change in diet to AID^10^. However the AID was not associated with serum inflammatory parameters (CRP, IL-6 and TNF-alpha)^10^.

The main characteristics of an AID are a plant-based whole-food diet, with a high proportion of fiber, antioxidants, and a balanced ratio of omega-3 and omega-6 fatty acids, with omission of sugar^10–14^. In this context it could be shown, that a low-carbohydrate diet (with a daily amount less than carbohydrates of 130 g) reduces levels of IL-6 and hsCRP as well as features of metabolic syndrome and improves the risk factors for heart disease^15,16^. Hujoel (2009) also identified the role of sugar as an gingivitis-increasing dietary component^11^. Moreover, there is growing evidence that lipid metabolism, in addition to excess carbohydrates, is also closely related to a pro-inflammatory metabolic state^17,18^. In this context, omega-6 and omega-3 polyunsaturated fatty acids are known as essential fatty acids that cannot be synthesized by the human body and are supplied through the diet. Omega-6 fatty acids are naturally found as linoleic acid (LA) and the metabolite arachidonic acid (AA) while omega-3 fatty acids are represented by alpha-linoleic acid (ALA) and the metabolites eicosapentaenoic acid (EPA) and docopentahexaenoic acid (DHA) among others^19^. Humans are able to convert LA to AA and ALA to EPA and DHA, but omega-6 fatty acids compete with omega-3 fatty acids for desaturation enzymes. Their metabolites lead to a variety of different effects and are, among other metabolites, precursors for pro- and anti-inflammatory series of eicosanoids and resolvins^20^. Excessive levels of omega-6 fatty acids leading to high ratios of omega-6:omega-3 PUFAs are associated with prothrombotic and pro-inflammtory effects contributing to obesity, diabetes, and atherosclerosis^21^. Furthermore, a diet rich in omega-6 fatty acids could be shown to increase the level of pro-inflammatory eicosanoids and oxidative stress and thus promotes periodontal inflammation^22,23^. Conversely, supplementation of marine omega-3 fatty acids was associated with a reduction of inflammatory surrogate markers. In a review by Chee et al., the intake of omega-3 fatty acids was found to improve clinical periodontal outcomes^24^, and this was confirmed by a recently published meta-analysis^13^.

Besides omega-3 fatty acids, a large number of other serum fatty acids may influence inflammatory processes. Saturated fatty acids were shown to significantly increase post-prandial dendritic cell circulation (as a sign of innate immunity) in healthy volunteers in contrast to omega-3 poly-unsaturated fatty acids^25^. In a study published in 2010, by Ramirez-Tortosa et al., the levels of a broader spectrum of fatty acids were analyzed with regard to periodontitis^26^. They found a positive correlation between levels of palmitic acid (C16:0) and saturated fatty acids and periodontal disease, in terms of pocket depth and clinical attachment level. The pro-inflammatory effect of palmitic acid on increased periodontal bone resorption was confirmed in an animal model^27^. Palm oil is a good source of palmitic acid and one of the vegetable fats most commonly used for industrial food production worldwide^28^.

While the described studies are showing promising associations between several fatty acids and periodontitis, there is lack of information regarding their effect on gingivitis. Furthermore, though dietary interventions on gingivitis have clinical relevant effects, there is still missing information which macro- and micronutrients are responsible for this. To the best of the authors’ knowledge, no study to date has reported on the serum fatty acid profile in gingivitis patients during the course of a dietary intervention. The present explorative trial aimed to further investigate a comprehensive fatty acid profile with regard to periodontal parameters in patients with gingivitis on an AID.

## Methods

### Ethics approval and consent to participate

This clinical trial adhered to the principles of the Declaration of Helsinki on human experimentation (World Medical Association Declaration of Helsinki 18) and was carried out in accordance with Good Clinical Practice. The Ethical Committee of the Faculty of Medicine at the University of Freiburg verified this trial with a positive vote (EK No. 8/16). Patient recruitment took place in the Department of Operative Dentistry and Periodontology, Faculty of Medicine, University of Freiburg, Germany. All enrolled participants signed an informed consent form as well as a data privacy statement. The study was registered in an international trial register (German Clinical Trial Register number DRKS 00009888, Registration date 15/02/2016). This report followed the criteria of the CONSORT statement for randomised clinical trials 19 (see CONSORT checklist, related manuscript file). Participants received 100 Euros for participation.

### Study design

This exploratory study further investigated the serum fatty acids profile in gingivitis patients at baseline and at the end from a previous randomised clinical trial^10^. This was conducted at the Department of Operative Dentistry and Periodontology, Faculty of Medicine, University of Freiburg, Germany. In addition to a quantitative analysis of various fatty acids, the association of these fatty acids on clinical periodontal parameters was analysed.

### Participants

All participants of the previous study needed to meet the following inclusion criteria:

- Gingivitis as defined by a Löe & Silness^29^ gingival index (GI) ≥ 0.5 or BOP > 10%^30^
- Following a common high carbohydrate intake > 45%^16^
- Age ≥ 18 years

The exclusion criteria were as follows:

- Periodontitis as defined by a Community Periodontal Index of Treatment Needs by Ainamo et al.^31^ ≥ 3 or 4, with a score of ≥ 3 or 4, for ≥ 2 sextants
- Smoking
- Severe or life-threatening illnesses
- Intake of antibiotics within 6 months before the start of or during the study period
- Use of drugs influencing gingival inflammation or bleeding (e.g., anticoagulants, cortisone)
- Carbohydrate- or insulin-related diseases (e.g., diabetes)
- Pregnancy or lactation

### Intervention

The participants were recruited consecutively, allocated by a statistician (KV) stratified by the values of the plaque index (PI), and assigned to either the experimental group (n = 15) or the control group (n = 15); the clincial examiner (MG) was blinded to group allocation throughout the whole study (single-blinded study). For the first 2 weeks (baseline), both groups were not given any additional nutritional recommendations, other than continuing their pro-inflammatory dietary pattern (PID). The experimental group was placed on an AID for 4 weeks after 2 transitional weeks, whereas the control group was instructed to continue their habitual PID for another 6 weeks. The duration of 4 weeks for the experimental group was chosen based on other comparable studies^5,12^. The recommended diet for the experimental group was low in processed carbohydrates and animal products, and rich in fibers, omega-3 fatty acids (by a daily portion of fish or an omega-3 supplement), vitamin C and D, antioxidants, and plant nitrates^10^. All participants of the experimental group received an individual 30-min dietary counselling with a dentist specialised in nutritional medicine (JPW, CT). After a verbally assessment of the habitual diet, the dietary counselling informed about both recommended and non-recommended nutrient and suggested examples for wholes menus and product preparation. All questions and possible challenges were discussed with the participants. Furthermore, the participants were given the chance to contact the dietitian for further questions. The AID compilation was based on studies showing reduced gingivitis and/or systemic inflammation by certain dietary interventions^5,11,13,32,33^. For the full recommendations patients received see Supplementary File 1. The participants in the experimental group were given two “transitional” weeks in order to empty all pro-inflammatory and non-recommended products in their household. All participants were told to stop their interdental hygiene (like interdental brushing or flossing) throughout the study period in order not to mask possible signs of inflammation. However, all participants had to continue tooth brushing with a manual tooth brush. All participants were instructed to fill out a 24 h‐dietary diary for 1 week at the second, fifth and eighth week in order to check their compliance. Clinical parameters, such as the PI^34^, GI^29^, pocket probing depth (PD), bleeding on probing, and periodontal inflamed surface area (PISA)^35^ were examined at baseline and after 4 weeks of intervention, using a digital periodontal software (Parostatus, Parostatus.de GmbH, Berlin, Germany). For further information regarding the study and the AID protocol, see Woelber et al.^10^.

### Fatty acid analysis of serum lipids

Serum examinations of fatty acids were performed by a specialised laboratory (MVZ Clotten, Freiburg, Germany). Serum was obtained by using a standardised blood collection system that contained a gel (S-Monovette, 7.5 ml Z-Gel, Sarstedt, Germany) for the separation of about 3 mL of serum from clotted blood cells by centrifugation. The isolated serum was stored at < − 20 °C for a maximum of 5 days until analysis. The serum lipids were analysed as fatty acid methyl esters (FAMEs) by gas chromatography with flame ionisation detection (GC-FID). Therefore, the serum was treated with methanol/acetylchloride (20/1 v:v; 90 °C/90 min) to generate methyl esters. After cooling to room temperature, the preparation was neutralised with carbonate solution and the FAMEs were extracted by vortex-shaking with hexane. The organic phase was separated and injected (4 µL) into the GC-FID system (7890A, Agilent Technologies, Santa Clara, California, USA). We focused our work on the analysis of the entire serum fatty acid components because both the esterified and the free fatty acids seem to change synergetic under dietary conditions^36^. Free fatty acids were not measured separately.

Chromatographic separation was performed using an SP-2560 capillary GC column (100 m × 250 μm × 0.2 μm; Supelco Analytical, Division of Sigma-Aldrich, St. Louis, USA**)** using a temperature gradient from 140 to 240 °C, for 35 min. For quantification of the different fatty acids, the rare, natural nonadecanoic acid (C19:0 for C10‒C20:2) and pentacosanoic acid (C25:0 for C20:3‒C24) served as internal standards, which were added to the serum before preparation. A certified FAME mix (C4‒C24) and commercial docosapentaenoic acid (C22:5w3) were used as reference materials. All fine chemicals were purchased from Sigma-Aldrich (Sigma-Aldrich, St. Louis, USA). The serum concentrations were calculated by separate two-point-calibration curves measured within the corresponding analysis series for each analysed fatty acid component (Table 2).

### Statistical analysis

The sample size calculation was carried out for the main study^10^. It was calculated that with a sample size of 15 patients per group, a difference of 5% between the groups in the change of the mean value of GI with a SD of 5 can be detected with a power of 80%. These specifications correspond to an effect size of 1.1. After pseudonymisation, the participant data were transmitted to the statistician. The statistician (KV) performed a randomisation stratified by the measured plaque index using the statistical software STATA (version 14.2, Stata Corp, College Station, Texas, USA).

The primary outcome of this substudy was a quantitative description of the serum fatty acid profile, while secondary outcomes were the effects of serum parameters on GI, PI, PD, BOP, and PISA. All outcomes were in response to a 4-week period of an AID for the test group and a PID for the control group. For descriptive analysis, the mean and standard deviation were computed. A paired t-test was used to check for changes in the outcome variables from the baseline to the end of the study within each group. To analyse differences in the changes between the groups, t-tests were applied. Linear regression models adjusting for group effects were used to estimate both the association of serum parameters on clinical parameters (GI, PI, PD, BOP, and PISA) as well as the changes in serum parameters on changes in clinical parameters. Despite the many statistical tests performed, no correction was made for multiple testing, as the investigation was exploratory in nature. All analyses of clinical and serum data were performed using STATA 14.2 (StataCorp, College Station, Texas, USA).

## Results

**Figure 1:**
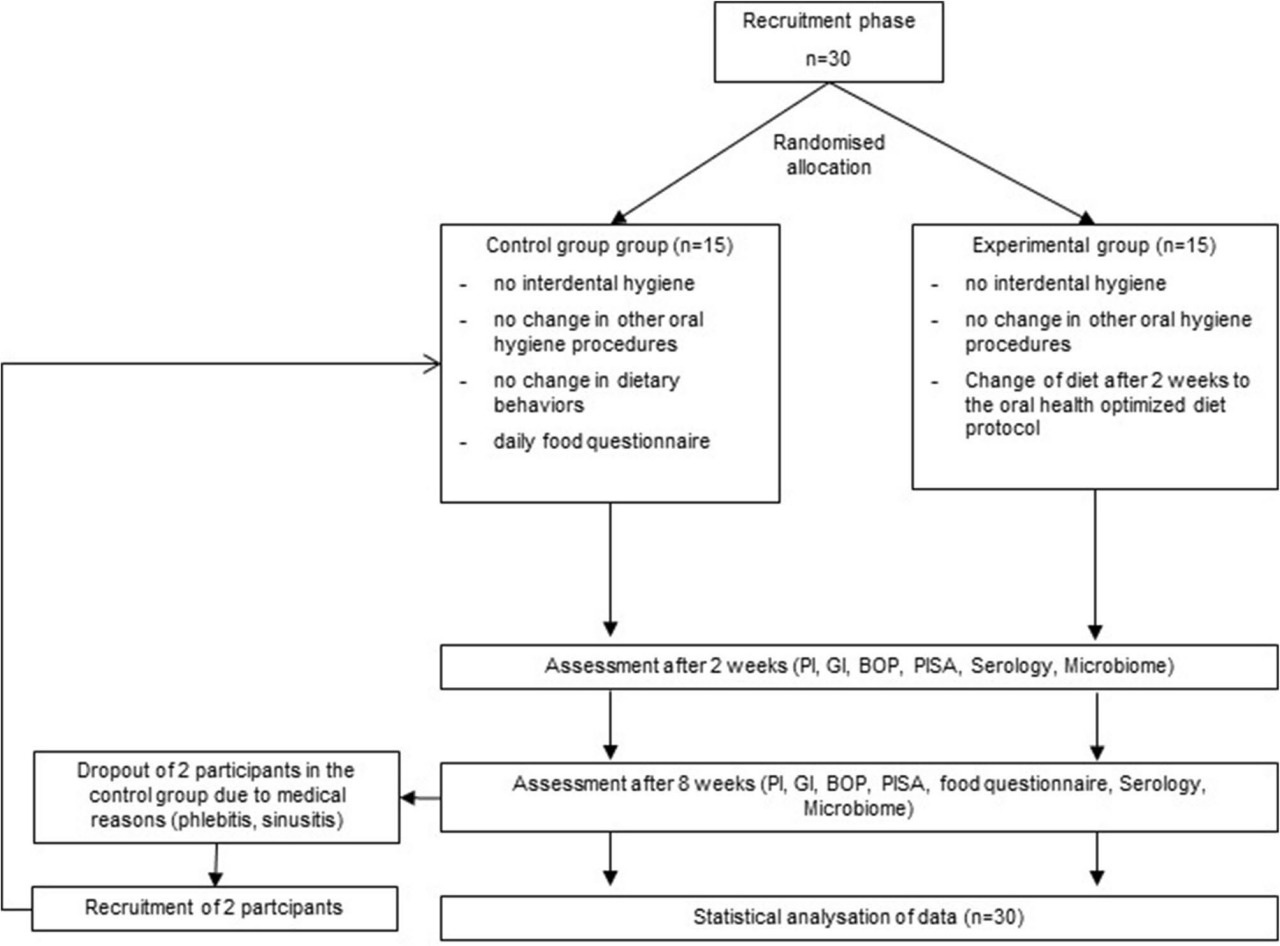
Study flow chart.

Thirty out of 38 participants completed the investigation. Six had to be excluded due to a diet that differed from the PID criteria. There were two dropouts in the control group because of the development of either phlebitis or sinusitis (Fig. 1). Patient recruitment and clinical procedures took place between October 2016 and August 2017. All participants were residents of Freiburg, Germany. Demographic and anthropometric data are shown in Table 1. None of the participants were obese. The groups did not differ at baseline for body weight and BMI. Throughout the study period, the mean weight loss in the experimental group was about 1.5 kg (p = 0.007) while the control group did not show significant changes in body weight (p = 0.310, Δintergroup: p = 0.006) (Table 1). There was a significant higher reduction of GI for the experimental group compared to the control group over the study period (experimental group BL 0.92 [0.14], end of the study 0.61 [0.29], p < 0.001; control group BL 0.83 [0.22], end of the study 0.74 [0.18], p = 0.007; Δintergroup: p = 0.03). Both groups showed a significant reduction for PI over the study period (experimental group BL 0.56 [0.27], end of the study 0.48 [0.13], p = 0.016; control group BL 0.57 [0.19], end of the study 0.48 [0.12], p = 0.006; Δintergroup: p = 1). For BOP there could be found a significant reduction for the experimental group only (experimental group BL 30.35 [11.07], end of the study 23.55 [13.61], p = 0.031; control group BL 28.39 [13.32], end of the study 27.09 [10.03], p = 0.529; Δintergroup: p = 0.864). For PISA no significant changes were found over the study period (experimental group BL 315.27 [148.68], end of the study 252.37 [151.78], p = 0.111; control group BL 270.5 [140.97], end of the study 286.0 [114.02], p = 0.345; Δintergroup: p = 0.60). For detailed information on clinical data see^10^.

**Table 1:** Demographic and anthropometric data of the AID and PID group.

Group	Age, years	Female (%)	Male (%)	Body weight in kg		Body mass index in kg/m^2^	
				BL	End	BL	End
AID group (n = 15)	27.2 (± 4.7)	9 (60%)	6 (40%)	71.7 (± 11.5)	70.2 (± 11.0)	23.5 (± 2.3)	23.3 (± 2.9)
PID group (n = 15)	33.7 (± 13.1)	8 (53.3%)	7 (46.7%)	72.5 (± 16.3)	73 (± 15.9)	23.0 (± 2.1)	24.7 (± 4.94)

Data are given as means (± standard deviation) or absolute numbers (percent).

*BL* baseline.

The dietary change of the AID group resulted in a significant lower caloric intake (p < 0.001), a significant reduction of carbohydrate intake (p < 0.001), a significant increase of fiber intake (p < 0.001), a significant reduction in total fat intake (p < 0.001), but a significant increase in PUFAs (p < 0.001). Furthermore, the salt intake dropped significantly (p < 0.001). The diet of the control group did not change significantly^10^.

### Intragroup differences

Significant intragroup differences between the baseline and study completion were found only in the experimental group, who showed a higher polyunsaturated fatty acids (PUFA):saturated fatty acids (SFA) ratio, Omega 3-Index ([EPA + DHA] × 100/TFA) and AA:DGLA-Index at the end of the study than at baseline. Both groups showed a baseline Omega-3-Index in the pro-inflammatory and near-inflammatory range, respectively. Additionally, levels of myristic acid, palmitoleic acid, gamma-linolenic acid, and lignoceric acid were significantly lower in the experimental group than in the baseline (Table 2).

**Table 2:** Mean values (standard deviations) of serum fatty acids for the experimental and control group at different time points and results of intragroup and intergroup differences.

Fatty acids [normal range]	Group	Baseline	End	Intra-p-value^a^	Inter-p-value [∆exp vs. ∆control]^b^
**Middle chain fatty acids**					
Capric acid C10:0 [n/a]	Exp	4.51 (2.26)	3.2 (1.25)	0.089	0.502
	Control	5.09 (2.03)	4.55 (2.36)	0.534	
Lauric acid C12:0 [n/a]	Exp	15.32 (18.69)	5.6 (4.73)	0.066	0.196
	Control	12.65 (9.20)	10.55 (6.29)	0.506	
**Long chain fatty acids**					
Myristic acid C14:0 [< 70 mg/L]	Exp	52.63 (39.32)	24.95 (9.73)	0.023*	0.155
	Control	54.89 (23.10)	47.62 (23.71)	0.423	
Myristoleic acid C14:1 [< 10 mg/L]	Exp	3.81 (1.67)	2.89 (1.03)	0.101	0.862
	Control	4.56 (1.71)	3.8 (2.76)	0.296	
Palmitic acid C16:0 [350–870 mg/L]	Exp	794.2 (437.43)	550.27 (104.79)	0.062	0.269
	Control	770.87 (250.63)	696.33 (209.49)	0.422	
Palmitoleic acid C16:1 [15–68 mg/L]	Exp	61.91 (21.29)	43.88 (21.78)	0.003**	0.254
	Control	67.95 (19.70)	61.24 (32.36)	0.434	
Stearic Acid C18:0 [200–910 mg/L]	Exp	386.07 (523.12)	166.8 (32.10)	0.135	0.354
	Control	287.33 (299.69)	220.27 (77.16)	0.438	
Oleic acid C18:1ω9c [440–1200 mg/L]	Exp	620.87 (122.59)	565.07 (130.28)	0.201	0.524
	Control	648.73 (132.19)	634.13(196.62)	0.767	
Linoleic acid C18:2ω6c [750–1300 mg/L]	Exp	988.53 (231.84)	962.27 (344.53)	0.730	0.679
	Control	1017.13 (159.05)	953.2 (166.17)	0.227	
α-Linolenic acid C18:2ω6c [4–15 mg/L]	Exp	28.12 (11.08)	38.29 (23.64)	0.053	0.063
	Control	29.58 (15.08)	26.97 (9.28)	0.573	
γ-Linolenic acid C18:3ω6 [5–23 mg/L]	Exp	14.63 (5.52)	11.12 (3.70)	0.018*	0.348
	Control	17.88 (6.79)	16.72 (10.24)	0.588	
Arachidonic acid C18:3ω6 [20–400 mg/L]	Exp	285.2 (87.70)	260.93 (78.07)	0.073	0.992
	Control	344.4 (90.34)	319.93 (82.91)	0.139	
Eicosapentaenoic acid C18:3ω6 [3–36 mg/L]	Exp	31.62 (10.49)	31.19 (17.94)	0.932	0.643
	Control	48.29 (19.67)	44.37 (19.04)	0.488	
**Very long chain fatty acids**					
Behenic acid C22:0 [20–45 mg/L]	Exp	15.85 (11.48)	10.02 (2.77)	0.100	0.632
	Control	14.21 (7.87)	10.29 (2.78)	0.091	
Erucinic acid C22:1ω9 [< 4 mg/L]	Exp	2.59 (2.94)	2 (0.93)	0.404	0.299
	Control	1.73 (0.71)	2.37 (3.62)	0.507	
Docosadienoinic acid C22:1ω9 [n/a]	Exp	2.15 (0.44)	2.37 (0.83)	0.470	0.096
	Control	2.24 (0.62)	1.8 (0.67)	0.093	
Docosapentaeic acid C22:1ω9 [< 25 mg/L]	Exp	16.75 (3.21)	14.87 (3.28)	0.0828	0.833
	Control	19.61 (5.94)	18.11 (5.27)	0.315	
Docosahexaeic acid C22:6ω3 [25–80 mg/L]	Exp	89.71 (41.03)	93.79 (32.02)	0.536	0.124
	Control	114.77 (34.85)	103.87 (36.99)	0.139	
Lignoceric acid C24:0 [10–50 mg/L]	Exp	13.21 (5.29)	8.80 (3.10)	0.016*	0.390
	Control	18.86 (16.44)	10.49 (3.58)	0.068	
Nervonic acid C24:1ω9 [20–70 mg/L]	Exp	18.36 (3.97)	19.62 (1.05)	0.314	0.044*
	Control	20.87 (5.86)	18.39 (4.43)	0.076	
**Trans fatty acids**					
Elaidic acid C18:1ω9t	Exp	7.07 (6.17)	4.37 (2.08)	0.125	0.187
	Control	5.79 (3.62)	6.25 (4.43)	0.79	
trans-Linoleic acid C18:2ω6t	Exp	2.37 (0.73)	2.3 (0.97)	0.824	0.763
	Control	2.08 (0.81)	1.89 (0.61)	0.41	
**Sums and ratios**					
Total fatty acids (TFA)	Exp	3558.73 (1114.36)	2975.33 (590.14)	0.093	0.518
	Control	3616.13 (733.15)	3306.33 (781.58)	0.262	
Saturated fatty acids (SFA)	Exp	1298.53 (1043.43)	776.8 (143.39)	0.087	0.307
	Control	1178.13 (594.36)	1010.07 (308.66)	0.388	
Monounsaturated fatty acids (MFA)	Exp	715.27 (138.51)	641.4 (141.22)	0.114	0.488
	Control	750.93 (149.28)	726.8 (224.50)	0.670	
Polyunsaturated fatty acids (PUFA)	Exp	1535.53 (345.44)	1550.67 (325.65)	0.825	0.199
	Control	1679.47 (266.40)	1561.80 (308.86)	0.142	
Ratio PUFA:SFA	Exp	1.56 (0.54)	2.00 (0.18)	0.010*	0.046*
	Control	1.58 (0.34)	1.61 (0.27)	0.812	
Omega 3 fatty acids (all)	Exp	168.87 (49.93)	181.07 (50.37)	0.346	0.111
	Control	215.07 (62.46)	195.57 (56.28)	0.204	
Omega 6 fatty acids (all)	Exp	1366.6 (307.48)	1369.6 (294.12)	0.960	0.176
	Control	1464.4 (225.39)	1351.33 (245.64)	0.081	
Ratio Omega 6 (all): Omega 3 (all)	Exp	8.34 (1.61)	7.90 (1.84)	0.234	0.305
	Control	7.21 (1.69)	7.34 (1.75)	0.760	
Omega 3-Index ([EPA + DHA]*100/TFA; serum index)	Exp	3.66 (1.31)	4.20 (1.22)	0.044*	0.111
	Control	4.61 (1.36)	4.44 (0.88)	0.641	
Ratio AA : EPA	Exp	9.756 (3.96)	11.06 (5.98)	0.420	0.659
	Control	7.97 (3.28)	8.39 (3.70)	0.731	
Ratio AA : DGLA	Exp	4.36 (1.16)	5.18 (1.18)	0.035*	0.187
	Control	4.80 (1.35)	4.96 (1.25)	0.620	

The displayed values represent fatty acids with significant changes; normal range is given where available: normal ranges were provided by the executing laboratory.

*p < 0.05, **p < 0.01 (all fatty acid concentrations are given in mg/L).

^a^Paired t-Test to compare baseline vs. week 8 within each group for each fatty acid.

^b^Two sample t-Test comparing group changes ∆exp vs. ∆control for each fatty acid.

### Intergroup differences

The only differences in changes between baseline and study completion between the groups were the changes in the nervonic acid level decreasing in the control group and the PUFA:SFA ratio, which increased significantly more in the experimental group than in the control group (Table 2).

### Regression analysis

#### Analysis of all baseline and final values

At baseline, PD was significantly positively associated to nervonic acid (p = 0.029). GI and BOP did not show any significant association with fatty acids at baseline. At this time, capric acid, gamma linolenic acid, and trans linoleic acid showed a significant association to PI (Table 3). An increase of about 1 mg/L in capric acid was associated with an increase of 0.04 in PI. While capric acid and gamma linolenic acid were positively associated, trans-linoleic acid was negatively associated with PI. At the end of the study period, several fatty acids were negatively associated with PD (oleic acid, total fatty acids, monounsaturated fatty acids, polyunsaturated fatty acids, and omega-6). Furthermore, EPA was positively associated with GI, AA:DGLA with PI, and elaidic acid with PISA (Table 3).

**Table 3:** Overview of significant associations of serum parameters to clinical parameters of all participants with corresponding regression coefficients based on linear regression analysis.

	Regression coefficient					*p*				
	PI	GI	PD	BOP	PISA	PI	GI	PD	BOP	PISA
Capric acid C10:0	*0.040*					*0.012*				
Elaidinic acid C18:1ω9t					*14.510*					*0.046*
Oleic acid C18:1ω9c			** < − 0.001**					**0.010**		
Trans linoleic acid C18:2ω6t	*− 0.100*					*0.027*				
Gamma linoleic acid C18:3 ω6	*0.013*					*0.022*				
EPA C20:5ω3		**0.005**					**0.046**			
Nervonic acid C24:1ω9			*0.029*					*0.002*		
Total fatty acids			** < − 0.001**					**0.039**		
Monounsaturated fatty acids			** < − 0.001**					**0.011**		
Polyunsaturated fatty acids			** < − 0.001**					**0.030**		
Omega-6 fatty acids			** < − 0.001**					**0.036**		
AA:DGLA	**0.039**					**0.047**				

The displayed values represent fatty acids with significant associations only. Values in italics show associations at baseline; values in bold show associations at the end of the study.

*PI* plaque index, *GI* gingival index, *PD* pocket probing depth, *BOP* bleeding on probing, *PISA* periodontal inflamed surface area.

#### Analysis of overall differences from baseline to study end

The difference in PD (ΔPD) in the total study cohort was significantly associated to the change between final and baseline values (Δ) for linoleic acid, Δcapric acid, ΔALA, and Δlignoceric acid (Table 4).

**Table 4:** Significant association of fatty acid levels to differences in probing depth (PD).

Fatty acids	Regression coefficient	*p*
	PD	PD
Capric acid C10:0	0.030	0.035
Linoleic acid C18:2ω6c	0.006	0.013
α-Linoleic acid C18:3ω3	0.006	0.013
Lignoceric acid C24:0	0.007	0.045

Significant association of fatty acid levels to differences in probing depth (PD) from baseline to the end of the study, for all participants.

#### Analysis of differences between the experimental and control groups

For the experimental group, ΔDPA was negatively associated with ΔPI, while ΔALA, Δlinoleic acid, Δoleic acid, and Δmonosaturated fatty acids associated positively with a higher ΔBOP and ΔALA, and Δlinoleic acid associated with a higher ΔPISA. The control group showed significant associations for ΔPI, ΔGI, ΔPD, and ΔBOP (Table 5). Interestingly, ΔALA was associated positively with ΔBOP in the experimental group, but negatively with ΔBOP in the control group. The majority of serum fatty acids did not show any significant differences among the different groups (Table 2).

**Table 5:** Significant correlations of differences for the experimental and the control group.

Fatty acids	Regression coefficient					*p*				
	PI	GI	PD	BOP	PISA	PI	GI	PD	BOP	PISA
Oleic acid C18:1ω9c				*0.039*					*0.029*	
Linoleic acid C18:2ω6c			**0.007**	*0.323*	*1.704*			**0.038**	*0.035*	*0.018*
α-Linoleic acid C18:3ω3			**0.007**	*0.323*	*4.629*			**0.038**	*0.035*	*0.018*
				**− 0.219**					**0.062**	
Arachidonic acid C20:4ω6	**0.001**		**0.002**			**0.030**		**0.024**		
Erucinic acid C22:1ω9		**− 0.031**		**− 1.139**			**0.005**		**0.044**	
Docosapentaeic acidC22:5ω3	*− 0.022*					*0.040*				
Lignoceric acid C24:0			**0.009**					**0.019**		
Nervonic acid C24:1ω9	**0.019**					**0.018**				
MFA				*0.037*					*0.025*	

The displayed values represent fatty acids with significant correlations only. Values in italics show correlations of differences for the experimental group; values in bold show correlations of differences for the control group.

## Discussion

The aim of the present study was to investigate changes in the fatty acid profile of patients with gingivitis and their association with clinical periodontal parameters after a 4-week AID intervention, as compared to a control group following a PID. While changes in the Omega 3-Index, PUFA:SFA ratio, and AA:DGLA ratio were higher in the experimental group, the results showed no major differences in the fatty acid profiles between the experimental and control groups. Furthermore, serum inflammatory parameters like CRP, IL-6 and TNF-alpha were not found to be reduced^10^. Thus, no clear association between serum fatty acids and anti-inflammatory processes were observed. Some reports have suggested that serum fatty acids can act as biomarkers of diet^37,38^. However, the increase in the PUFA:SFA ratio for the experimental group over the study period as well as the finding that this ratio was significantly higher in the experimental than in the control group, shows an anti-inflammatory tendency associated with the AID. Other scientific studies also came to this conclusion^39–41^. Murumalla et al. found evidence of the anti-inflammatory effects of PUFAs (EPA, DHA and oleic acid) in overweight individuals^39^. Rocha et al. found that SFAs stimulate pro-inflammatory genes, in contrast to MUFAs and omega-3 PUFAs, which have a more anti-inflammatory effect^40^. Yang et al. reported that a high n6-PUFA/SFA ratio was beneficial for cardiovascular diseases^41^. However, the data of the present study, suggested that the increased ratio in the experimental group was rather due to a reduction in SFAs than to an increase in PUFAs. In this context it should also be noted that the generic term PUFA here includes several fatty acids besides omega-3 and omega-6 fatty acids contributing to a wide variety of effects.

Final nervonic acid levels were significantly higher than baseline levels in the experimental group. The role of nervonic acid as a sign of pro- or anti-inflammatory processes is controversial in the literature. Some authors consider it to have an anti-inflammatory effect, since nervonic acid serves as a precursor of antioxidative serum plasmalogens^42^. An increase in nervonic acid could also simply indicate an increased intake of oleic acid (18:1w9, e.g., in olive, rape, or sunflower oils; “seed oils”), since nervonic acid is an elongation product of oleic acid. Muralidharan et al. established that reduced nervonic acid and plasmalogen levels were linked to metabolic syndrome, and peroxisomal dysfunction^42^. Szczuko et al. also investigated the influence of diet on plasma fatty acids, particularly nervonic acid^43^. In their study, which included women with polycystic ovary syndrome, they attributed a pro-inflammatory effect to nervonic acid. However, the inflammatory orientation of nervonic acid (pro-inflammatory or anti-inflammatory) may also be dependent on the tissue investigated.

PD was significantly associated with levels of several fatty acids (oleic acid, TFA, MFA, PUFA, and Omega-6). In this context, serum fatty acids do not appear to be a clear indicator of anti-inflammatory processes. During the AID, the Omega-3 Index did not increase to a more anti-inflammatory range, while the AA:DGLA ratio showed a significant increase. This effect could be explained by the results of a study in which GLA was supplemented in humans and led to increased circulating levels of both DGLA and AA^44^. Additionally, due to the weight loss in the experimental group, it can be speculated that there was a reduction in visceral fat tissue. During this process, the release of pro-inflammatory fatty acids into the bloodstream is conceivable and could explain the ambiguous distribution of the detected free fatty acids in the serum and their associations. On the other hand, the weight reduction observed in the experimental group in this study could also change the fatty acid profile. Here, Δ5-desaturase activity and a decrease in DGLA (C20:3, n-6) may play a role^45^. In particular, the higher AA:DGLA Index seems confusing in this context^44^. The anti-inflammatory effects of carbohydrate metabolism by a low-sugar, high-fiber diet seemed to have a much more immediate impact on clinical parameters in terms of lower BOP or reduced pocket depths, within days^11,46^. One could speculate that it takes longer for fats to have measurable effects on inflammatory processes, and these may be missed in a short study period.

Ramirez-Tortoza et al. compared the plasma fatty acid profile of periodontitis patients with that in a control group without periodontitis^26^. Significant differences were found between the groups, with the periodontitis group showing higher levels of TFA, PUFA, n-6 PUFA, MFA, SFA, and specific fatty acids in the periodontitis group. Interestingly, the levels of all tested fatty acids were higher, and mostly significantly higher, in the periodontitis group than in the control group^26^. Thus, it cannot be concluded that specific fatty acids could serve as inflammatory markers for periodontal disease. In this context it has to be taken into account that rather, periodontitis seems to be associated with dyslipidemia, as some authors claim^26,47,48^. Furthermore, Ramirez-Tortoza and colleagues also evaluated correlations of fatty acids with clinical periodontal parameters. They found that PD and clinical attachment loss showed significant positive correlations with the levels of palmitic acid, TFA, SFA, PUFA n-6, and PUFA. In the present study, TFA, SFA, PUFA n-6, and MFA were negatively associated with PD at the end of the study period. This might emphasize the differences in the lipid profile of gingivitis patients as compared to periodontitis patients.

Beside these interesting associations between fatty acids and the periodontal status, the mechanism of action seems still discussable and there is a profound need of explaining studies. A major challenge is the broad variance of fatty acids, the interaction between single nutrients and systemic influences (like due to weight loss) on the one side. On the other side, even the pathomechanism of gingival inflammation harbors several influences, primarily between a highly diverse microbiome and host immune system^49^. Based on these different influences, one might speculate that the main action of fatty acids will be based on their influence on inflammation, while inflammatory processes on the gingiva will promote an exudation of gingival crevicular fluid, which is nutritional requirement for gingivitis-associated, inflammophilic pathogens^6,7^. However, a diet rich in fatty acids might also lead to less plaque and to local anti-bacterial effects^50^.

There are several limitations of the study which have to be discussed. Due to the explorative character of this study with analysis of a secondary outcome parameter (fatty acids) the study was not sufficiently powered for this outcome parameter which is a major limitation. Furthermore, the study was limited by the short period (4 weeks) of dietary intervention. This duration was selected on the basis of other comparable studies^5,12^. While some metabolic processes seem to become clinically measurable very rapidly when altering carbohydrate consumption, such as BOP, a reduction of inflammation in terms of serum fatty acid levels might only benefit from a long-term dietary change^24^. Another limitation might be that daily oral hygiene in terms of tooth brushing was still performed, even though interdental cleaning had been suspended for the study period.

Therefore, future studies are essential to investigate the effect of an AID further, over a longer period of time, and with different biomarkers and surrogate parameters (serum, gingival crevicular fluid, clinical, composition of the sulcus fluid, and microbiological parameters) in conditions with and without oral hygiene limitations. Regarding the dietary intervention future studies should consider a dietary analysis based on the Dietary Inflammatory Index (DII) and a direct observation of food intake Also a lipidome analysis of resolvins and other metabolites of omega-3 fatty acids could be considered for further investigation.

## Conclusions

While the AID protocol led to a significant and clinically relevant reduction in periodontal inflammation, the fatty acid profile remained essentially unchanged in patients with gingivitis. Although some fatty acids showed individual associations with clinical parameters related to inflammation, there seems to be no clinically relevant association with gingivitis over the 4-week study period. Accordingly, other mechanisms (like carbohydrate-related effects or micronutrients) might be related to the short-term gingivitis-reducing effects of an AID.

## Supplementary Information

Supplementary Information.

## Data Availability

The datasets used and/or analysed during the current study are available from the corresponding author on reasonable request.

## Abbreviations

Δ: Difference
AA: Arachidonic acid
AID: Anti-inflammatory diet
BL: Baseline
BMI: Body mass index
BOP: Bleeding on probing
DGLA: Dihomo-γ-linolenic acid
DHA: Docosahexaenoic acid
EPA: Eicosapentaenoic acid
HMT: Host modulation therapy
GI: Gingival index
MFA: Monounsaturated fatty acid
PD: Pocket probing depth
PI: Plaque index
PID: Pro-inflammatory dietary pattern
PISA: Periodontal inflamed surface area
PUFA: Polyunsaturated fatty acid
SFA: Saturated fatty acid
TFA: Trans-unsaturated fatty acid
