# Supplementary material for: An exploratory study on the role of serum fatty acids in the short-term dietary therapy of gingivitis
Source: Sci Rep. 2022 Mar 7;12:4022. doi: 10.1038/s41598-022-07989-5 (PMC8901712; doi:10.1038/s41598-022-07989-5)
Supplement: Supplementary file 1 — Supplementary Information. [file 41598_2022_7989_MOESM1_ESM.pdf]

Excerpt from the original study information for participants

-translated from German-

Dear study participants,
Here you will receive detailed information on the study in written form.

The dietary change includes the following features:
The goal is a micronutrient-rich diet, rich in vitamins, minerals, trace elements, antioxidants, secondary plant compounds and fibre.

This includes...
... omission of refined high-glycaemic carbohydrate foods. This includes pasta and bread, baked goods, sugar, honey, sweets, sweetened drinks such as cola, lemonade, juices or beer.

In the case of starchy vegetables, please ensure a reduced consumption of less than 130g/day. This applies for example to rice, potatoes, millet, quinoa, couscous, etc.

However: Please continue to consume fruits, vegetables, nuts, seeds and legumes (e.g. beans, lentils) as much as you want! Drink mainly water and/or unsweetened teas. Consume only low-glycaemic alcohol and that in moderation (such as dry wine).

Daily intake of:

- Vitamin C: Easily consumed through fruits and vegetables.
  Aim for at least 120 mg a day
  Contained e.g. in: 2 kiwis, 1 orange, 1 pepper (raw), 100g broccoli (raw)
  Please note that vitamin C is greatly reduced when heated.

- Omega-3 fatty acids:
  Target: >0.5 g linolenic acid, DHA/EPA per day.

Contained e.g. in: mainly in fatty fish (DHA, EPA) and 1 tablespoon linseed oil, 2 tablespoons linseed meal, chia seeds, 1-2 walnuts, walnut oil (linolenic acid).

- Antioxidants and secondary plant substances:

Contain e.g.: 2 cups of green tea, a handful of berries and other plant foods rich in colour,

1 pinch of turmeric, ginger, coffee without milk.

- Dietary fibre:

Contained e.g. in: plant foods (vegetables, salad, fruit, whole grains, nuts, seeds, legumes), prebiotic fibre such as onions, leeks, garlic, chicory - and corresponding reduction of animal products (meat, milk, etc.).

- Vitamin D:

1000 IU a day by tablet. This is not necessary on days with strong sunlight exposure (e.g. sunbathing).

Avoid omega-6 fatty acids and trans fats:

Contained e.g. in: deep-frying fats, sunflower oil, grape seed oil, egg yolk, butter, peanuts/peanut oil.

Reduce saturated fatty acids as much as possible:

Contained e.g. in: animal products from factory farming (meat, dairy products, cheese, etc.)
